# Transgender health issues addressed in research on telehealth use: a scoping review

**DOI:** 10.3389/fsoc.2024.1371524

**Published:** 2024-11-25

**Authors:** Susanne Gahbauer, Daniela Haluza

**Affiliations:** ^1^Department of Social and Preventive Medicine, Medical University of Vienna, Vienna, Austria; ^2^Department of Science and Technology Studies, University of Vienna, Vienna, Austria; ^3^Department of Environmental Health, Medical University of Vienna, Vienna, Austria

**Keywords:** transgender persons, transgender health, scoping review, COVID-19, telehealth, transgender men, transgender women

## Abstract

Telehealth is a valuable tool for reminding transgender-persons to undergo HIV testing, hormone injections, and voice training. Despite increased awareness of the unique health needs of transgender-individuals in recent years, effectively addressing their concerns remains challenging. The COVID-19 pandemic has negatively affected socioeconomic status, mental health, and access to gender-affirming treatment. To better understand how individuals and their specific health issues are addressed in telehealth solutions, we conducted a scoping review using PubMed and Scopus, spanning from 2000 to 2021. We screened eligible articles following the PRISMA checklist, extracted the data, and performed a thematic analysis of 100 reports. For control purposes, we double-checked the ratings during the screening and coding. Our results show that telehealth is primarily used for healthcare access, sexual and reproductive health, and mental health. Since 2020, the overall number of publications has greatly increased, with only nine reports explicitly referring to COVID-19-related challenges. The latter relate exclusively to clinical purposes such as healthcare access. We found that transgender-women were researched more often than transgender-men, particularly in the field of sexual and reproductive health research. Some studies included groups other than transgender-persons, such as parents or clinicians, who might be relevant for advancing telehealth use. The review’s findings highlight the need for more research that considers the diversity of transgender-groups and the adapted use of diverse technological tools beyond pandemics and public health crises.

## 1 Introduction

Telehealth is a healthcare delivery method using telecommunication technology such as video conferencing to provide healthcare services and information remotely (Haluza et al., 2020), allowing individuals to connect with healthcare professionals from the comfort of their own homes or on the go when applying mobile applications. It is currently used in a wide range of healthcare services, including consultations, diagnosis, treatment, monitoring, and education (Haluza and Jungwirth, 2018). Telehealth is particularly useful for patients in remote or rural areas, those with mobility or transportation issues, and those experiencing difficulty accessing traditional healthcare services—such as transgender-persons being confronted with stigma, discrimination, and violence while seeking healthcare (Smith et al., 2018; Reisner et al., 2016). It has the potential to improve access to healthcare, increase patient engagement and satisfaction, and reduce costs (Radix et al., 2022; Restar et al., 2021; Russell et al., 2022; Shipherd et al., 2016). The COVID-19 pandemic profoundly impacted healthcare systems worldwide, with vulnerable groups, such as transgender-persons, being particularly affected (Restar et al., 2021). During a crisis, when many people stay at home and have limited in-person interactions, telehealth has become increasingly popular (Doraiswamy et al., 2020).

Despite recent efforts to address transgender-persons’ unique healthcare needs, the provision of adequate care remains challenging. In complex interdisciplinary settings, in which transgender-persons and healthcare workers interact, telehealth has been used to support healthcare needs, such as reminding users to conduct HIV tests or voice training (Jarrett et al., 2021; Wang et al., 2020; McGregor et al., 2023). Familiarity with the needs of transgender-individuals has grown in recent years, particularly in developed Western nations, and progress has been made. However, addressing specific health issues is challenging, given that people living under different political and social conditions depend on their home country and socioeconomic status (Asaad et al., 2020). Thus, the terminology (e.g., hijra, kathoey, travesties, and waria) used to describe transgender-populations worldwide across different socio-cultural settings is constantly evolving (Reisner et al., 2016).

Moreover, transgender-individuals face significant health disparities and stressors, which can lead to negative health outcomes if not addressed adequately (MacCarthy et al., 2015). This is translated in higher rates of mental health issues, such as depression and anxiety in the transgender-community than in the general population (Pinna et al., 2022). Transgender-persons may also face barriers to healthcare, such as a lack of knowledge among healthcare providers about their specific health needs or a lack of insurance coverage for gender-affirming treatments (Reisner et al., 2016), which encompass a range of social, psychological, behavioral, and medical interventions “designed to support and affirm an individual’s gender identity” (World Health Organization (WHO), 2024) when it conflicts with the gender they were assigned at birth.

Therefore, exploring this population’s health issues in telehealth research is crucial as little is known about the services that are used to provide health care and information remotely, also beyond the clinic and other medical institution (Roy et al., 2022). Putting telehealth for transgender-persons at the center of this review is of immense importance, as several studies have shown that the COVID-19 pandemic has negatively affected transgender-individuals´ lives worldwide, including their socioeconomic conditions, access to gender affirmation services, and mental health outcomes (Restar et al., 2021; Jarrett et al., 2021; Wang et al., 2020). Puckett et al. (2019) highlighted the importance of community support for individuals and the negative impact of its absence on mental health during the COVID-19 pandemic. Delays in accessing gender-affirming healthcare, including hormonal treatment and surgical care, detrimentally affected individuals’ health status additionally (van der Miesen et al., 2020). Hence, the focus of this research was the period from 2000 to the COVID-19 pandemic.

According to Radix et al., technological solutions for healthcare purposes can be categorized as telehealth or mobile health (Radix et al., 2022). As previously demonstrated, technology meant to facilitate healthcare, such as practitioner-patient communication, might also produce disparities, such as disparities between younger (i.e., “digital natives”) and older generations (i.e., “digital migrants”) (Haluza and Hofer, 2020; Naszay et al., 2018), as younger individuals are highly engaged online and well connected within online communities (Russell et al., 2022; McGregor et al., 2023; Mintz et al., 2022) compared to older populations.

Telehealth has emerged as an increasingly important tool for healthcare delivery, particularly in the wake of the COVID-19 pandemic (Monaghesh and Hajizadeh, 2020). However, the extent telehealth solutions address people’s specific health concerns remains unclear, as recent reviews have focused on aspects of accessibility and the quality of telehealth-enabled healthcare services (McGregor et al., 2023; Mintz et al., 2022). Our current review aimed to address this research knowledge gap and provide a more conclusive understanding of the health issues addressed in research on telehealth use. In this context, our research explored what kind of technological solutions are in use and investigated, what health issues they address, and how transgender-persons are addressed in scientific literature across multiple disciplines.

## 2 Methods

The PRISMA (Preferred Reporting Items for Systematic Reviews and Meta-Analyses) framework supported a clear presentation of data sources, screening stages, and inclusion criteria, aligning with the scoping nature of our study by providing a structured and comprehensive overview of the available literature. Therefore, we employed the PRISMA framework to structure the data selection and reporting processes to ensure transparency and reproducibility in study selection (Tricco et al., 2018).

### 2.1 Eligibility criteria

For our literature review, we included articles that investigated telehealth focusing on or intended for transgender-persons, published since 2000 in peer-reviewed journals in German and English, and presented original data. Aside gender-affirming treatments we also included all the other specific health concerns of transgender-persons. Review articles as well as articles in other languages and those without a full text were not included in our literature search.

### 2.2 Literature search

We searched *PubMed* and *Scopus* databases in cooperation with the Library of the Medical University of Vienna. In the first query, we also included the search term *trans*, resulting in the exclusion of more than 30,000 records from further processing. The search terms used were transsex*, *transgender*, *eHealth*, *mHealth*, *telehealth*, *mobile applications,* and *apps.* Since research on this topic began predominantly in the early 21st century, the search encompassed more than two decades of publication data (2000–2021). Consequently, our analysis included information gathered from January 1, 2000, to December 31, 2021.

### 2.3 Selection process

Before initiating the literature search, we thoroughly examined online academic sources to determine whether a similar review had already been undertaken. Although we conducted a scoping review, we used the Preferred Reporting Items for Systematic Reviews and Meta-Analyses (PRISMA) checklist for reporting purposes (Page et al., 2021). Since transgender-persons were not clearly specified or the focus was limited to sexual practice in several studies, we investigated telehealth solutions focused on transgender-persons rather than other groups of people, even if they may intersect.

The term “Intersectionality” was coined by the racial theorist Kimberlé Crenshaw (1989) and describes how identities and movements are discussed as separated and constructed if they were standing alone. Intersectionality, however, describes how “systems of inequality intersect to ´create´ unique dynamics and effects “(TGEU (Trans Europe and Central Asia), 2024) which shape individuals’ experiences, opportunities, and forms of discrimination or privilege (Hammarström and Hensing, 2018). For this review, we considered the following systems of inequality: gender, race, ethnicity, sexual orientation, gender identity, disability, and class. Nevertheless, we did not consider studies that recruited along sexual practice, as numerous studies have recruited men who have sex with men but reported transgender-persons as a separate group, thus frequently reduced being transgender to sexual practices.

### 2.4 Data collection and analysis

Data were extracted using Excel (version 16.6). Our data grid contained the following variables: consecutive number; first author; article type; year of publication; rating within the screening (three to five stars records); COVID-19 pandemic virus-related (yes/no); study type and methods used; country and region; addressing transgender-persons in the study (e.g., transgender, trans-women); abbreviations used (e.g., TGNB, meaning *trans-gender and non-binary* and describes people whose gender does not match that assigned to them at birth); number and age of transgender-persons included in the study; intersectionality (e.g., young, black, or recently incarcerated); diagnosis, treatment, and intervention; control and comparison; and study outcome.

Recognizing the profound influence of diverse writing styles on vulnerable populations, we conscientiously chose to employ a hyphen in “trans-” during the analysis of the specific groups under consideration in our studies. This deliberate choice reflected our commitment to sensitivity and precision in addressing language nuances, particularly when discussing and representing vulnerable communities (Stryker et al., 2008). This analytical concept allowed us to analyze how transgender-persons intersect with other groups and categories (e.g., women, men, sex, gender, or young/old, ethnicity) to reflect on diversity aspects and aspects of intersectionality without being reductive towards transgender-individuals.

We used thematic analysis for establishing themes to describe the different health issues we identified in our review. Coding followed a five-step approach, assigning single codes to each category (Braun and Clarke, 2006; Braun and Clarke, 2014). We also conducted control steps in terms of intercoder reliability.

## 3 Results

### 3.1 Study selection

**Figure 1:**
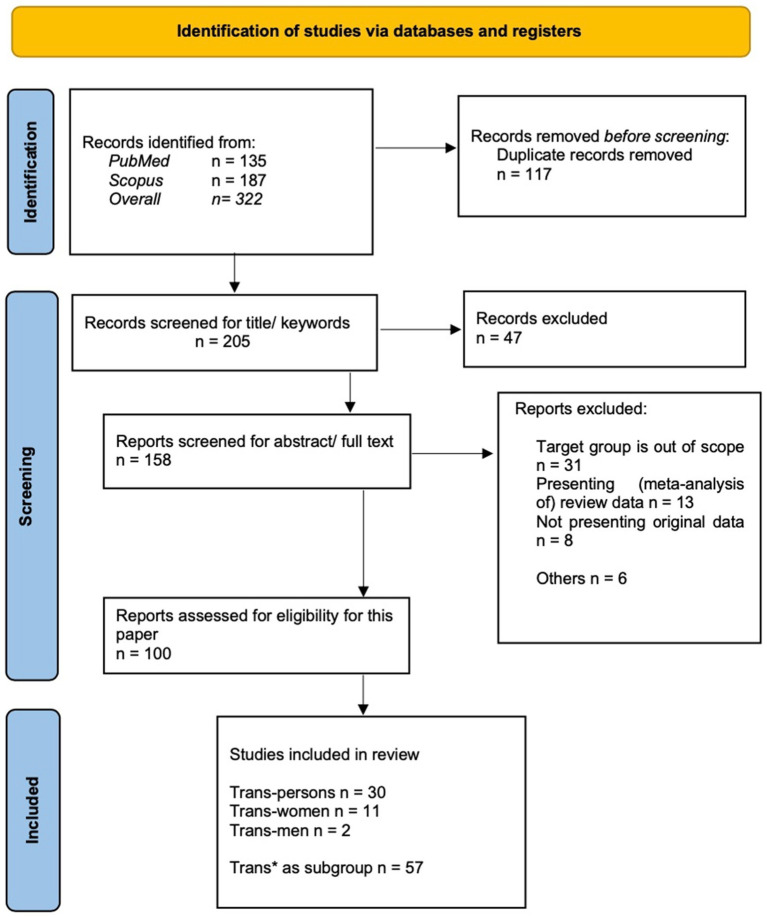
Preferred reporting items for systematic reviews and meta-analyses (PRISMA) checklist.

In total, 100 records were included in the final analysis. Overall, we identified 322 articles: 187 from Scopus and 135 from PubMed (Figure 1). The search records were exported to EndNote (Version X9.3.3) for screening purposes. After removing 117 duplicates, 205 records were screened by title and keywords. Each record was initially screened on the 5-star scale in Endnote, and 3- to 5-star records were considered for further analyses. For this purpose, we focused on the key term “telehealth” and excluded all the technological tools, that did not focus on health issues, such as geographical networking apps or technological tools whose focus was not the health of transgender-persons. To be precise about the inclusion thresholds, we checked 2- and 3-stars ratings twice. Furthermore, 47 records were excluded from the analysis because they were outside the scope of this study, such as not being peer-reviewed articles, being published in a Spanish-language journal, or not adequately covering our literature search. Ultimately, 158 records were downloaded for retrieval.

After screening the abstracts and full-text of these records, 57 reports were excluded from further analysis, as 31 records addressed groups that were outside the study scope (e.g., sex workers or people living with HIV not specified as transgender-persons), 21 records did not present original data, and 6 were excluded for other reasons (e.g., presenting an English abstract but the article text in Turkish, or outside the scope of the study). The screening and exclusion of records was double-checked by the authors.

### 3.2 Study characteristics

We found that the first article on telehealth for transgender-persons was published in 2009. Since then, the number of articles published a year ranged between 1 and 13. In 2020, with the outbreak of the COVID-19 pandemic crisis, this number increased: more than a half of the reports were published since then (*n* = 61) even though just a few of them (*n* = 9) directly refer their findings to the pandemic situation (Table 1). Geographically, the majority of the studies was conducted in the US and Canada (*n* = 68). Only four of these publications referred to the COVID-19 virus pandemic explicitly. Only one of the ten studies conducted in Asia did not refer to sexual health, such as testing for sexually transmitted infections, minimizing risks of sexual behavior, or health promotion in general (Table 1).

**Table 1:** Details of reviewed publications (*n* = 100).

Category	Number of articles
Year of publication	
2009	1
2010	1
2011	1
2014	2
2015	3
2016	5
2017	6
2018	13
2019	7
2020	24
2021	37
Geography	
Africa	2 (0)*
North-America	68 (4)
South- and Middle America	5 (0)
Europe	11 (4)
Asia	10 (0)
Australia and New Zealand	3 (0)
Global Sample	1 (1)
Subsamples	
Trans- x sexual orientation x cis-	11
Trans-women x sexual orientation/practice	34
Trans-men x sexual practice x intersectionality	1
Sexual and gender minorities	4
Acronyms, such as LGBT (QIA+)	7

*Numbers in parentheses: Studies related to COVID-19 per region. LGBT (QIA+): Lesbian, Gay, Bisexual, Trans (Queer, Intersex, Asexual, + all others).

More than a half of the publications (*n* = 57) presented transgender-persons as a subsample in a larger sample, sometimes even including only a few persons in samples larger than 1,000 (Table 1). In these samples, transgender-persons were rather addressed as “trans-women” or “transgender-women” (*n* = 33) than as “trans-men,” who were only addressed in one report (*n* = 1). Eleven articles examined “trans-(gender-) men and -women” or “trans-gender” persons/individuals equally as cis persons (male or female) and/or lesbian/gay/bi persons/individuals or as those living with HIV. A further eleven reports recruited participants along minority groups or acronyms, such as “sexual and gender minorities” or “LGBT*” (i.e., lesbians, gays, bisexuals, and transgender). Hence, transgender-persons were part of the study samples but not the focus of these studies (Table 1).

### 3.3 Telehealth for transgender-persons, transgender-women, and transgender-men

Telehealth can be used for both clinical and nonclinical purposes (World Health Organisation, 2010). *Clinical use* refers to patient management and diagnostics, whereas *nonclinical use* refers to education, mentoring, supervision, research, and health promotion. Hence, we sorted our study outcomes into clinical and nonclinical telehealth and observational studies (Tables 2–7, respectively). Table 2 shows the clinical telehealth tools for healthcare (e.g., access), sexual health, mental health, and health communication as well as the specific target population described in each of the included articles.

**Table 2:** Clinical telehealth tools and health issues, namely healthcare (access), sexual health, mental health, and health communication.

Tool		Target Population	Number of Articles
Health issue I: healthcare (access)			
Telemedicine such as video visits (Russell et al., 2022; Gava et al., 2021; Silva et al., 2021)	gender affirming care; routine clinical care	trans-gender youth and caregivers	2
E-Mail-based (eConsultation of the Veterans Affairs [VA])* (Shipherd et al., 2016; Kauth et al., 2015; Blosnich et al., 2019)	LGBT health	trans-veterans and interdisciplinary clinicians	3
E-Consultation *(Rubicon MD)* (Potapov et al., 2021)	Hormone treatment	trans-gender and non-binary	1
Health issue II: sexual health			
SMS-, app-based and e-Coaching *(Tech Step)* (Benkeser et al., 2020; Reback et al., 2020)	HIV prevention	trans-gender youth and young adults	2
Home HIV/STI testing supplemented with telehealth-based consulting (Sharma et al., 2019)	quantification of HIV/STI testing rates	trans-gender youth	1
Health issue III: mental health			
App *(TransLife)* (Dubov et al., 2021)	Suicidal ideation	trans-persons	1
Web-based via *Zoom (AFFIRM)* (Craig et al., 2021)	Cognitive behavioral theory (CBT) group intervention	trans-persons	1
Website (Pankey et al., 2021; Fraser, 2009)	Promotion of gender-affirming tele-psychology	trans-gender and gender diverse	1
Health issue IV: health communication			
E-Consultation *(Champlain BASE)* (Singh et al., 2021)	Primary care provider and trans-gender care specialist communication	trans-gender	1
Web-based (Ross and Castle, 2017)	Patient-practitioner communication	trans-patients	1

*Tools with a proper name are written in italics.

**Table 3:** Clinical telehealth tools: research purpose, methodologies and sample sizes, settings.

Authors, publication date, title	Research purpose/question	Methodologies	Sample size, setting
Health issue I: healthcare (access)			
Gava et al. (2021): Mental Health and Endocrine Telemedicine Consultations in Transgender Subjects During the COVID-19 Outbreak in Italy: A Cross-Sectional Web-Based Survey	Access to healthcare and effect on mental health and endocrinological care	Cross-sectional web- based survey, 3 validated questionnaires (IES, BDI-II, SF-12)	*N* = 108, clinical trial
Russell et al. (2022): Increasing Access to Care for Transgender/Gender Diverse Youth Using Telehealth: A Quality Improvement Project	Increase access to gender-affirming care by expanding telehealth without compromising communication, privacy, and patient satisfaction	Patient needs assessment, 9-items evaluation survey of video visits	*N* = 69 and *N* = 91, academic multidisciplinary pediatric center
Silva et al. (2021): Usability of Virtual Visits for the Routine Clinical Care of Trans Youth during the COVID-19 Pandemic: Youth and Caregiver Perspectives	Families’ perspectives on the usability of virtual visits for routine care for trans youth during the COVID-19 pandemic	Online study, validated telehealth usability questionnaire (TUQ)	*N* = 28 trans youth and *N* = 69 caregiver, gender clinic in tertiary children’s hospital
Kauth et al. (2015): Teleconsultation and Training of VHA Providers on Transgender Care: Implementation of a Multisite Hub System	Evaluating teleconsultation and training program for transgender care (to train and educate front-line clinicians, expand clinical capacity)	Evaluation study, participation in at least 12 of 14 program sessions	*N* = 5, interdisciplinary clinical teams in a 14-session, 7-months program; *N* = 13, participants in evaluation study
Shipherd et al. (2016): Nationwide Interdisciplinary E-Consultation on Transgender Care in the Veterans Health Administration	Establishing a nationwide interdisciplinary e-consultation program for transgender care	Feasibility study, multimethod approach	*N* = 303, teleconsultations for *N* = 230 veterans
Blosnich et al. (2019): Utilization of the Veterans Affairs´ Transgender E-Consultation Program by Health Care Providers: Mixed-Methods Study	Describing experiences with the program, reasons for non-use and ways to improve its use	Mixed methods, semi-structured interviews for users and online survey for non-users	*N* = 4 urban and *N* = 11, rural provider; *N* = 53 non-users
Potapov et al. (2021): Electronic Consultations as an Educational Tool to Improve the Care of Transgender Patients in Primary Care	Exploring the impact of e-consultations submitted to a TGNB specialist panel on primary care clinicians’ experience and on their education on TGNB-related topics	Document analysis of de-identified data from the RubiconMD platform	*N* = 4 TGNB specialists in Community Health Center in NY that responded to N = 74 e-consultations over 24 months
Health issue II: sexual health			
Benkeser et al. (2020): Design and Analysis Considerations for a Sequentially Randomized HIV Prevention Trail	Testing the efficacy of different combinations of interventions (TechStep includes a SMS system, web application and electronic counseling)	Formative research, sequential multi-arm randomized trail (SMART)	*N* = 250 users
Reback et al. (2020): Technology-Based Stepped Care to Stem Transgender Adolescent Risk Transmission: Protocol for a Randomized Controlled Trial (TechStep)	Testing improvement in taking sexual risk or PrEP uptake	Randomized controlled trial	*N* = 54, on- and offline recruiting
Sharma et al. (2019): Variations in Testing for HIV and Other Sexually Transmitted Infections across Gender Identity among Transgender Youth	Quantifying HIV and other STI testing levels and examining variations in testing levels across trans-men, trans-women and nonbinary individuals	Randomized trial, multivariable logistic regression	*N* = 186, HIV home testing supplemented with telehealth-based consulting
Health issue III: mental health			
Dubov et al. (2021): Development of a Smartphone App to Predict and Improve Rates of Suicidal Ideation among Transgender Persons (TransLife): A Qualitative Study	Beta testing the usability of an evidence-informed suicide prevention phone app: preliminary data on user engagement and satisfaction and assessing the feasibility of completing EMAs (mood logs) within the app	Pilot study with exploratory research approach: qualitative methods combining naturalistic app use, focus groups and semi-structured phone interviews, 2 validated questionnaires for user experience SUS and SUPR-Q	*N* = 16, respondent-driven sampling
Craig et al. (2021): Adapting Clinical Skills to Telehealth: Applications of Affirmative Cognitive-Behavioral Therapy with LGBTQ+ Youth	Presenting key considerations for CBT delivery via telehealth, detail clinical skills and strategies; describing the adaptation approach through a case study; offering guidance to support the adaptation of clinical skills to a virtual environment	Case study, online group sessions	*N* = 1
Pankey et al. (2021): Gender-Affirming Telepsychology During and After the COVID-19 Pandemic: Recommendations for Adult Transgender and Gender Diverse Populations	Promoting the delivery of gender-affirming telepsychology; highlighting clinical issues observed by health service psychologists	Clinical vignette	*N* = 1, high volume gender clinic
Fraser (2009): Etherapy: Ethical and Clinical Considerations for Versions 7 of the World Professional Association for Transgender Health’s Standards of Care	Background and explanatory material for the potential inclusion of etherapy in Version 7 of the Standards of Care	Literature overview, case study	*N* = 1, a Saudi-based American male-to-female trans-person
Health issue IV: health communication			
Singh et al. (2021): Evaluation of an Electronic Consultation Service for Transgender Care	The impact of an electronic consultation (eConsult) service between primary care providers and transgender care specialists	Retrospective mixed methods analysis, identification of six major themes	*N* = 62 electronic consultations
Ross and Castle (2017): A Culture-Centered Approach to Improving Healthy Trans-Patient-Practicioner-Communication: Recommendations for Practitioners Communicating with Trans Individuals	trans-individuals’ lived experiences with practitioners and the types of advice they suggested be provided to practitioners treating trans-persons to improve the patient-practitioner relationship	Semi-structured qualitative interviews, thematic analysis	*N* = 13, convenience snowball sample

**Table 4:** Non-clinical telehealth tools and health issues.

Tool		Target population	Number of articles
Health issue I: sexual and reproductive health (research)			
Dating apps (*Tinder*, GrindR*, ao) (Albury et al., 2021; Masullo and Coppola, 2021)	experiences of sexual healthcare; safety while using apps; HIV/ STI testing; online perpetuation of exclusion	app users	2
Video-chat counselling *(Project Moxie)* (Stephenson et al., 2020)	HIV testing rates	trans-gender youth	1
Web-based e-service *(Sexual Health London [SHL])* (Day et al., 2021)	Epidemiological data; HIV/STI testing	trans-gender and non-binary	1
Webinar on COVID-19 and sexual and reproductive health and rights (MacKinnon and Bremshey, 2020)	Impact of COVID-19 on trans-persons; health	trans-persons and other groups	1
Web-based *s*ervice (*GenderGP*) (Rogers et al., 2020)	Positive and negative determinants of gamete storage	trans-persons and gender diverse patients	1
Health issue II: drug use			
Geo-enabled smart phone apps (McQuoid et al., 2021)	Multiple drug use	trans-person with autism spectrum disorder	1
Health issue III: healthcare (Research)			
Dating app *(Hornet)* (Jarrett et al., 2021)	Increasing access to gender-affirming resources	trans-gender and non-binary	1
*Google Android Panel* (Sell et al., 2015)	Epidemiological data on sexual orientation, gender identity and sex assigned at birth	trans-persons	1

^*^Tools with a proper name are written in italics.

**Table 5:** Research on non-clinical telehealth tools: research purpose, methodologies, and sample sizes, settings.

Authors, publication date, title	Research purpose/question	Methodologies	Sample size, setting
Health issue I: sexual and reproductive health (research)			
Albury et al. (2021): Not your Unicorn: Trans Dating App User’s Negotiations of Personal Safety and Sexual Health	Exploring app use and healthcare in the context of the interdisciplinary field of “digital intimacies”	Interviews and workshops, creative (visual) research methods	*N* = 14, mailing lists of sexual health organizations and social media accounts
Masullo and Coppola (2021): Scripts and Sexual Markets of Transgender People on Online Dating Apps: A Netnographic Study	User data, influence of dating apps on defining processes related to gender expressivity and sexual script construction, constitution of spaces that meet emotional and sexual needs, discriminatory dynamics	Netnographic study combining different online/offline qualitative techniques	observation of *N* = 300 users´ profiles, n = 30 semi-structured interviews
Stephenson et al. (2020): Project Moxie: Results of a Feasibility Study of a Telehealth Intervention to Increase HIV Testing Among Binary and Nonbinary Transgender Youth	Developing an intervention that provides home-based HIV self-testing coupled with video-chat counseling	Randomized feasibility study	*N* = 202, online recruiting
Day et al. (2021): Beyond the Binary: Sexual Health Outcomes of Transgender and Non-Binary Service Users of an Online Sexual Health Service	Comparing sexual health outcomes of TNB and cisgender users of London’s online sexual health service	epidemiological study, univariable logistic regression analysis	N = 119,329 registrants, n = 504 TNB
MacKinnon and Bremshey (2020): Perspectives from a Webinar: COVID-19 and Sexual and Reproductive Health and Rights	How sexual and reproductive health and rights (SRHR) are affected during the COVID-19 pandemic, especially in vulnerable social groups	Summary from a 100 min discussion on trans-persons and cross-cutting areas	*N* = 1,555 participants from 116 countries
Rogers et al. (2020): A Retrospective Study of Positive and Negative Determinants of Gamete Storage in Transgender and Gender-Diverse Patients	Formally categorizing the reasons trans-gender people store and do not store gamete prior to hormone treatment	Quantitative and qualitative data, analysis of electronic medical records	*N* = 3,667, recruiting via telemedicine service GenderGP
Health issue II: drug use			
McQuoid et al. (2021): Exploring Multiple Drug Use by Integrating Mobile Health and Qualitative Mapping Methods – An Individual Case Study	Assessing multiple drug use and its unique patterns, intentions, and social contexts	Mixed methods approach, geo-enabled smartphone survey data and qualitative mapping interview method	*N* = 1
Health issue III: healthcare (research)			
Jarrett et al. (2021): Gender-Affirming Care, Mental Health, and Economic Stability in the Time of COVID-19: A Multi-National, Cross-Sectional Study of Transgender and Nonbinary People	Impact of COVID-19 and subsequent control measures on gender-affirming care, mental health, and economic stability	Global, cross-sectional study, mental health indicator PHQ-4, Poisson regression models	*N* = 964, data from COVID-19 Disparities Survey
Sell et al. (2015): The Utility of an Online Convenience Panel for Reaching Rare and Dispersed Populations	Investigating the utility of a large nonprobability online panel to conduct rapid population assessments of sexual and gender minorities	Epidemiological study, using similar data from two-population based surveys (NHIS and NESARC)	*N* = 34,759, Google Android Panel

**Table 6:** Observational research on telehealth tools and health issues.

Tool		Target Population	Number of Articles
Health issue I: hormone treatment			
Web-based and social media (Sequeira et al., 2020)	Gender affirming healthcare (access and hormone prescription)	trans-persons	1
Telemedicine (Hertling et al., 2021)	Gender-affirming hormone treatment	trans-persons and gynecological endocrinologists	1
Health issue II: mental health			
Web-based (Fraser, 2009)	Online psychotherapy	trans-persons	1
Web-based or app-based (Trans Pathways)* (Perry et al., 2018)	Early intervention	trans- and gender diverse youth and parents and guardians	1

^*^Tools with a proper name are written in italics.

**Table 7:** Observational research on telehealth: research purpose, methodologies, and sample sizes, settings.

Authors, publication date, title	Research purpose/question	Methodologies	Sample size, setting
Health issue I: hormone treatment			
Sequeira et al. (2020): Transgender Youths´ Perspectives on Telehealth for Delivery of Gender-Affirming Care	Examining transgender youths´ interest in receiving gender-affirming care via telemedicine or through primary care with telehealth support	Online survey, bivariate analysis	*N* = 204, gender clinic
Hertling et al. (2021): Acceptance, Use, and Barriers of Telemedicine in Transgender Health Care in Times of SARS-CoV-2: Nationwide Cross-Sectional Survey	Analyzing the acceptance, use, and barriers of telemedicine in transgender healthcare in times of COVID-19	2 web-based cross-sectional surveys, descriptive statistics, regression analyses	*N* = 269 trans-persons and *n* = 202 gynecological endocrinologists
Health issue II: mental health			
Fraser (2009): Etherapy: Ethical and Clinical Considerations for Version 7 of the World Professional Association for Transgender Health’s Standards of Care	Background and explanatory material for the potential inclusion of etherapy in Version 7 of the Standards of Care	Literature overview, case study	*N* = 1, a Saudi-based American male-to-female trans-person
Perry et al. (2018): Online Interventions for the Mental Health Needs of Trans and Gender Diverse Young People	Exploring the mental health and care pathways	Online survey (Trans Pathways)	*N* = 859 trans-youth, *N* = 194 parents and guardians

Table 3 presents the clinical telehealth tools used for healthcare (access), sexual health, mental health, and health communication, along with each study’s research purpose, methodology, sample size, and study setting.

As shown in Table 2, most publications (*n* = 16) referred to the clinical use of telehealth, such as the tool AFFIRM (affirmative cognitive behavioral therapy group intervention), while nine focused on nonclinical use (Table 4), such as investigating experiences in sexual healthcare or creating epidemiological data from very large existing data samples (e.g., the Google Android Panel). Furthermore, four were observational studies measuring the acceptance of telehealth tools. The most frequently discussed topics were sexual and reproductive health issues (*n* = 9) and healthcare access and research (*n* = 8), whereas mental health was discussed in only five. The less-discussed topics were health communication (*n* = 2) and drug use (*n* = 1).

Table 5 shows details of the included research articles on nonclinical telehealth tools for sexual and reproductive health (e.g., research), drug use, and healthcare (research), along with each study’s research purpose, methodology, sample size, and study setting.

Regarding age groups, we found that in seven articles, transgender-persons were recruited in adolescence, ranging from 14 to 18 years. In four publications, other groups were included, such as parents and caregivers, gynecological endocrinologists, and interdisciplinary clinicians, but were each analyzed and reported as a separate group. In two articles (Albury et al., 2021; Masullo and Coppola, 2021) transgender-persons were recruited via the telehealth intervention itself, thus, through addressing transgender-persons among the group of transgender-dating app users. Using an intersectional approach (Hammarström and Hensing, 2018), two studies focused on transgender-veterans (Shipherd et al., 2016) or a transgender-person with autism spectrum disorder (McQuoid et al., 2021). Table 6 summarizes observational studies on telehealth for hormone treatment and mental health, along with the respective groups addressed in this study.

Table 7 shows details of the observational research articles on telehealth tools for hormone treatment and mental health along with each study’s research purpose, methodology, and sample size, and study setting.

We also clustered the reported telehealth solutions stratified by group (i.e., women and men). Table 8 shows telehealth tools focusing on transgender-women that encompassed sexual and reproductive health and voice work.

**Table 8:** Telehealth tools focusing on transgender-women and related health issues.

Tool		Target Population	Number of Articles
Health issue I: sexual and reproductive health (research)			
Web-based sub-study of a larger multisite prospective cohort (*LITE*) (Wirtz et al., 2021)	Digital epidemiological research; feasibility study on developing and implementing an online cohort to assess risks for HIV acquisition and other health experiences	trans-gender women	1
Web-based sex seeking platforms (websites and apps) (da Silva et al., 2020)	Factors shaping stigma; prevalence of web-based sex seeking	trans-gender women	1
Social media and technology-based networking platforms (Holloway et al., 2020)	Social network structure and composition; technology use patterns; accessing transgender health resources online	trans-gender women	1
Self-testing app (Akinola et al., 2021)	HIV prevention research	trans-gender women	1
App *(TransWomen Connected)* (Sun et al., 2020; Sun et al., 2019)	Sexual health promotion	trans-gender women	2
App *(Life Skills)* (Kuhns et al., 2021)	Group-based HIV prevention intervention (risk reduction)	young trans-gender women	1
Trans-gender women of color-specific telehealth intervention (Magnus et al., 2020)	Increasing access to care (HIV)	trans-gender women of color	1
Dating app *(GrindR)* (Lloyd and Finn, 2017)	Trans-feminist Foucauldian-informed discourse analysis between (socio-historic) trans-authenticity, validation and sexualization	trans-women	1
Web-based *s*ervice (*GenderGP*) (Rogers et al., 2020)	Positive and negative determinants of gamete storage	trans-persons and gender diverse patients	1
Health issue II: voice work			
Voice app (Hawley and Hancock, 2021)	Voice modification	trans-gender women	1

^*^Tools with a proper name are written in italics.

Table 9 shows details of the studies focusing on transgender-women, along with each study’s research purpose, methodology, sample size, and setting.

**Table 9:** Studies focused on transgender-women: research purpose, methodologies, and sample sizes, settings.

Authors, publication date, title	Research purpose/question	Methodologies	Sample size, setting
Health issue I: sexual and reproductive health (research)			
Wirtz et al. (2021): Digital Epidemiologic Research on Multilevel Risks for HIV Acquisition and Other Health Outcomes Among Transgender Women in Eastern and Southern United States: Protocol for an Online Cohort	Estimating incidence of HIV and other health outcomes among trans-gender women	Digital observational research, part of a larger multisite prospective cohort (LITE)	*N* = 580, convenience sampling
da Silva et al. (2020): Stigma and Web-Based Sex Seeking among Men Who Have Sex with Men and Transgender Women in Tijuana, Mexico: Cross-Sectional Study	Assessing the effectiveness of sex seeking platforms for engaging in HIV prevention and treatment services, determination of prevalence of web-based sex seeking and examining the factors that shape or are influenced by stigma	Utility study, interviewer-administered survey, multivariable logistic regression analysis	*N* = 561, using venue-based and respondent-based sampling
Holloway et al. (2020): Leveraging Social Networks and Technology for HIV Prevention and Treatment with Transgender Women	Developing health promotion strategies by using technology-based networking platforms	Qualitative methods, focus groups	*N* = 39 in *n* = 5 focus groups
Akinola et al. (2021): Perceived Acceptability and Feasibility on HIV Self-Testing and App-Based Data Collection for HIV Prevention Research with Transgender Women in the United States	Perceived acceptability of novel technologies for research purpose, particularly HIV self-testing and remote data collection through a mobile app	Qualitative methods, focus groups	*N* = 41 in *n* = 7 focus groups
Sun et al. (2020): A Sexual Health Promotion App for Transgender Women (Trans Women Connected): Development and Usability Study	Testing the usability and acceptability of the prototype Trans Women Connected mobile app	3-phase prototype development process, pre-and posttests, think-aloud protocols, open-ended questions	*N* = 16
Sun et al. (2019): Findings from Formative Research to Develop a Strength-Based HIV Prevention and Sexual Health Promotion mHealth Intervention for Transgender Women	Examining the sexual health needs in the context of their overall health and identifying overarching content framing strategies and content for a mobile health intervention	Formative research, focus groups and in-depth interviews	*N* = 37 in *n* = 4 focus groups and n = 20 interviews
Kuhns et al. (2021): A Uniquely Targeted, Mobile App-Based HIV-Prevention Intervention for Young Transgender Women: Adaptation and Usability Study	Adapting an efficacious group-based intervention to a mobile app Life Skills to reduce HIV risk; testing its acceptability and usability	user-centered design sessions, think-aloud protocol, recordings of screen activity, 2 validated questionnaires (PSSUQ and Health-ITUES)	*N* = 8 in design sessions, *n* = 10 in the usability trial
Magnus et al. (2020): Development of a Telehealth Intervention to Promote Care-Seeking Among Transgender Women of Color in Washington, DC	Informing the development of a group-specific telehealth intervention to increase access to HIV care	Formative qualitative semi-structured interviews, focus groups, thematic coding and content analysis	*N* = 22
Lloyd and Finn (2017): Authenticity, Validation and Sexualisation on Grindr: An Analysis of Trans Women’s Accounts	Trans-women’s talk of self/ other identifications in relation to their regular use of GrindR	Semi-structured in-depth interviews, Foucauldian-informed discourse analysis	*N* = 8
Rogers et al. (2020): A Retrospective Study of Positive and Negative Determinants of Gamete Storage in Transgender and Gender-Diverse Patients	Formally categorizing the reasons trans-gender people store and do not store gamete prior to hormone treatment	quantitative and qualitative data, analysis of electronic medical records	N = 3,667, recruiting via telemedicine service GenderGP
Health issue II: voice work			
Hawley and Hancock (2021): Incorporating Mobile App Technology in Voice Modification Protocol for Transgender Women	Exploring the effectiveness and acceptability of the innovative service delivery	Single-subject changing criterion design, outcome measures via acoustics, self and listener ratings of audio samples, program evaluation questionnaire	*N* = 4 completing a 10-week hybrid clinic-home voice intervention program

When examining telehealth solutions addressed to transgender-individuals, we found that more articles focused on transgender-women and that transgender-women were frequently included in study samples exclusively comprised of transgender-persons. Eleven studies discussed transgender-women (Kuhns et al., 2021; Lloyd and Finn, 2017), whereas only two studies discussed transgender-men (Table 10) (D'Angelo et al., 2021; Garcia et al., 2014). This discrepancy is particularly noticeable in studies on sexual and reproductive health. Ten tools addressed transgender-women and one transgender-men. Within the two groups, we also found unique research topics, such as post-surgery care for transgender-men and voice modification for transgender-women. Table 11 shows details of the reviewed studies focusing on transgender-men and each study’s research purpose, methodology, sample size, and setting.

**Table 10:** Telehealth studies focused on transgender-men.

Tool	Health Issues	Target Population	Number of Articles
Expansion of telehealth (D'Angelo et al., 2021)	Health state and access to gender affirming care	trans-men and men female at birth	1
Smart-phone app measuring flaccid and erect length and girth (Garcia et al., 2014)	Post-surgery-care (female-to-male gender confirming genital surgery)	trans-men	1

**Table 11:** Telehealth studies addressed at transgender-men: research purpose, methodologies, and sample sizes, settings.

Authors, publication date, title	Research purpose/question	Methodologies	Sample size, setting
D'Angelo et al. (2021): Health and Access to Gender-Affirming Care During COVID-19: Experiences of transmasculine individuals and men assigned female sex at birth	Impact of COVID-19 among trans-men; exploring how it has influenced their access to healthcare and overall health behaviors	Semi-structured interviews, inductive thematic analysis	*N* = 20, part of the together 5,000 study
Garcia et al. (2014): Overall satisfaction, sexual function, and the durability of neophallus dimensions following staged female to male genital gender confirming surgery: the Institute of Urology, London U.K. experience	Assessing patient genital-GCS related satisfaction, regret, pre/ post-op sexual function, genital preferences, and genital measurements post-op	Evaluation study, survey and app measuring flaccid and erect length and girth	*N* = 10 having undergone suprapubic phalloplasty; *N* = 15 having undergone radial artery forearm-flap phalloplasty, mono-center study

### 3.4 Telehealth for transgender-persons during the COVID-19 pandemic

Even prior to the COVID-19 public health crisis, researchers emphasized the importance of utilizing 21st-century technological innovations for groups with existing access (Asaad et al., 2020). As previously published (Gahbauer and Haluza, 2022), the COVID-19 public health crisis resulted in the uptake of telehealth interventions for transgender-persons. More than half of the articles were published since 2020, although not all referred to the COVID-19 pandemic. Within our sample, we found 17 articles explicitly referring to the COVID-19 public health crisis, of which nine that were analyzed in this chapter as they addressed transgender-persons, transgender-men, or transgender-women.

As Table 12 shows, the pandemic rather supported the use of telehealth for maintaining health and healthcare access (Russell et al., 2022), especially in the younger groups than in older groups. Further health issues addressed in the reports at hand were mental, endocrinological, and sexual health. The tool AFFIRM translated an effective behavioral group therapy into an online environment. The authors of the respective article also examined the telehealth learning effects resulting from the COVID-19 pandemic, such as the learning effects of online adaptations of clinical skills (Craig et al., 2021).

**Table 12:** Telehealth studies targeting transgender-persons regarding COVID-19 and health issues.

Tool		Target population	Number of articles
Health issue I: healthcare (access)			
Video-visit, telematic visit (Gava et al., 2021; Hertling et al., 2021)	Gender affirming care (e.g., endocrinological consultation)	trans-persons	2
Telehealth support like video-visits (Russell et al., 2022; Silva et al., 2021)	Gender affirming care; routine clinical care	trans-gender youth and caregivers	2
Dating app *(Hornet)* (Jarrett et al., 2021)	Increasing access to gender affirming resources	trans-gender and non-binary	1
Health issue II: mental health			
Website (Pankey et al., 2021)	Promoting gender-affirming psychology	trans-gender and gender diverse	1
Web-based via Zoom (*AFFIRM*) (Craig et al., 2021)	Cognitive behavioral theory (CBT) group intervention	trans-persons	1
Health issue III: hormone treatment			
Telemedicine (Russell et al., 2022)	Gender-affirming hormone treatment	trans-persons and gynecological endocrinologists	1
Health issue IV: sexual and reproductive health			
Webinar on COVID-19 and sexual and reproductive health and rights (MacKinnon and Bremshey, 2020)	Impact of COVID-19 on trans-persons’ health	trans-persons and other groups	1

^*^Tools with a proper name are written in italics.

Table 13 summarizes the telehealth tools that addressed transgender-persons and explicitly referred to the COVID-19 pandemic, along with each study’s research purpose, methodology, sample size, and setting.

**Table 13:** Telehealth tools explicitly referring to COVID-19 and their research purpose, methodologies, sample sizes, and settings.

Authors, publication date, title	Research purpose/question	Methodologies	Sample size, setting
Health issue I: healthcare (access)			
Gava et al. (2021): Mental Health and Endocrine Telemedicine Consultations in Transgender Subjects During the COVID-19 Outbreak in Italy: A Cross-Sectional Web-Based Survey	Accessing healthcare and effect on mental health and endocrinological care	cross-sectional web- based survey, 3 validated questionnaires (IES, BDI-II, SF-12)	*N* = 108, clinical trial
Hertling et al. (2021): Acceptance, Use, and Barriers of Telemedicine in Transgender Health Care in Times of SARS-CoV-2: Nationwide Cross-Sectional Survey	Analyzing the acceptance, use, and barriers of telemedicine in transgender healthcare in times of COVID-19	2 web-based cross-sectional surveys, descriptive statistics, regression analyses	*N* = 269 trans-person; *N* = 202 gynecological endocrinologists
Russell et al. (2022): Increasing Access to Care for Transgender/Gender Diverse Youth Using Telehealth: A Quality Improvement Project	Increasing access to gender-affirming care by expanding telehealth without compromising communication, privacy, and patient satisfaction	Patient needs assessment, 9-items evaluation survey of video visits	*N* = 69 and *N* = 91 academic multidisciplinary pediatric centers
Silva et al. (2021): Usability of Virtual Visits for the Routine Clinical Care of Trans Youth during the COVID-19 Pandemic: Youth and Caregiver Perspectives	Families’ perspectives on the usability of virtual visits for routine care for trans youth during the COVID-19 pandemic	Online study, validated telehealth usability questionnaire (TUQ)	*N* = 28 trans youth and *N* = 69 caregivers, gender clinic in a tertiary children’s hospital
Jarrett et al. (2021): Gender-Affirming Care, Mental Health, and Economic Stability in the Time of COVID-19: A Multi-National, Cross-Sectional Study of Transgender and Nonbinary People	Impact of COVID-19 and subsequent control measures on gender-affirming care, mental health, and economic stability	Global, cross-sectional study, mental health indicator PHQ-4, Poisson regression models	*N* = 964, data from COVID-19 Disparities Survey
Health issue II: mental health			
Pankey et al. (2021): Gender-Affirming Telepsychology During and After the COVID-19 Pandemic: Recommendations for Adult Transgender and Gender Diverse Populations	Promoting the delivery of gender-affirming telepsychology, highlighting clinical issues observed by health service psychologists	Clinical vignette	*N* = 1, high volume gender clinic
Craig et al. (2021): Adapting Clinical Skills to Telehealth: Applications of Affirmative Cognitive-Behavioral Therapy with LGBTQ+ Youth	Presenting key considerations for the delivery of CBT via telehealth; detailing clinical skills and strategies; describing the adaptation approach through a case study; offering guidance to support clinicians to adapt clinical skills to virtual environment	Case study, online group sessions	*N* = 1
Health issue III: hormone treatment			
Russell et al. (2022): Increasing Access to Care for Transgender/ Gender Diverse Youth Using Telehealth: A Quality Improvement Project	Increasing access to gender-affirming care by expanding telehealth without compromising communication, privacy, and patient satisfaction	Patient needs assessment, 9-items evaluation survey of video visits	*N* = 69 and *N* = 9, academic multidisciplinary pediatric center
Health issue IV: sexual and reproductive healthcare			
MacKinnon and Bremshey (2020): Perspectives from a Webinar: COVID-19 and Sexual and Reproductive Health and Rights	How sexual and reproductive health and rights (SRHR) are affected during the COVID-19 pandemic, especially in vulnerable social groups	Summary of a 100 min discussion on trans-persons and cross-cutting areas	*N* = 1,555 participants from 116 countries

## 4 Discussion

On a global scale, the demand for comprehensive and inclusive healthcare is rapidly increasing. This growing recognition highlights the importance of adopting a thoughtful approach that addresses various dimensions of transgender-persons´ lives and their specific health requirements (Pinna et al., 2022; Puckett et al., 2019; Kirby, 2016). Hence, we reviewed the pertinent literature to shed light on what kind of technological solutions are in use and investigated, what health issues they address, and how transgender-persons are addressed within tools and applications.

We found that the most often discussed health issue was healthcare (e.g., access), particularly during the COVID-19 public health crisis. Further important themes were sexual and reproductive health issues and mental health issues. The first is strictly tied to with sexually transmitted infectious diseases (STIs) and related to their prevention through risk management. When addressing mental health issues, we found several tools that were exclusively produced to support issues experienced by transgender-persons, such as Trans-Pathways and AFFIRM.

The review further showed that, especially for sexual health concerns, the clinic is extended through the use of technological tools that aren’t initially designed for health purposes. Dating apps, for instance, support the discussion of risky behavior and, have potential to collect epidemiological data on transgender-persons, amongst others. As other studies have shown, non-clinical tools such as social media, have great potential to support public health purposes, in particular health promotion efforts (Wernhart et al., 2019). On the other hand, it is difficult to select scientifically informed offers in non-clinical tools (de Vere and Linos, 2022).

The heterogeneity of groups and practices in the existing research limits the endeavor to provide accurate and appropriate guidance for transgender-specific initiatives. This also ties into the fact that some treatments are more relevant for only one particular group of transgender-persons: a masculinization of voice, for instance, is much easier to realize than a feminization, which could require surgical support (Gahbauer, 2019). Nevertheless, the aim of addressing a broadly defined population group while simultaneously reflecting on intersectionality might not adequately address group vulnerabilities. The concept of intersectionality is highly relevant to the current field of research on transgender-persons. By recognizing and addressing the interconnected nature of individuals’ identities and experiences, researchers can foster a more comprehensive understanding of their diverse health needs and develop inclusive approaches to meet those needs.

Notably, our literature review highlighted the emphasis on transgender-women within the realm of telehealth research. This was especially obvious in sexual health research (MacCarthy et al., 2015), in which sexual practice was seen as common ground. For further research, we would first suggest to address the data gap in transgender-men, and to consider their higher proportion of the sexual orientation ´gay´ and ´queer´ also in studies on telehealth (Reisner et al., 2023). And second, we assume that this fact is consistent with studies showing that women are objectified (Mol and Law, 2004; Gahbauer, 2015) and sexualized in medical practice and beyond (Lloyd and Finn, 2017). Hence, further investigation is necessary to determine whether transgender-women and transgender-men are treated differently in telehealth, not because one treatment is more relevant for one of the two groups but because of their association with the traditional binary categories of men/women and male/female. Similarly, a study by Rudin et al. demonstrated that even in completely different settings, transgender-women face greater discrimination than transgender-men (Rudin et al., 2023).

Since practitioners and other groups within and outside the healthcare system may not always feel comfortable discussing transgender-identity-related health issues, telehealth could be valuable for transgender-persons when not directly focusing on transgender-persons (Asaad et al., 2020; Craig et al., 2021). Within our data, we also found interventions aimed at practitioners, such as e-consultation programs connecting with a specialist in transgender-health, or involving parents of transgender-adolescents in an intervention program for mental health. The latter includes persons outside the healthcare system but might be crucial for the success of a medical intervention (Russell et al., 2022). Our review revealed a lack of established telehealth programs that targeted different populations working with transgender-persons. Yet, many of the included studies did not explicitly mention the COVID-19 public health crisis. Instead, it appeared to be a case of the “normative power of the factual,” such as adapting clinical skills to an online environment. We expect a steep increase in the implementation of established telehealth programs as a result of the COVID-19 pandemic in clinical contexts.

Despite the COVID-19 pandemic providing valuable insights for all stakeholders in the healthcare system, these insights have yet to translate into legal and policy progress (Coleman et al., 2022). Viewing telehealth as a risk, gender-affirming health services might become rather a question of economic power rather than of subjective body experience (Jarrett et al., 2021; McGregor et al., 2023; van der Miesen et al., 2020). Hence, for improving telehealth offers for transgender-persons it might be necessary to foster cooperation between industries and healthcare systems in order to provide state-of-the-art health technology and scientifically informed health services to all transgender-persons.

Due to the wide range of health issues, tools, and in particular methods presented in our data, conducting a quantitative meta-analysis was not possible. Therefore, we provided detailed information on individual reports. The diversity of the reports was reflected in the various settings where transgender-groups were recruited, including clinical settings, community centers, and through the use of apps or at local venues, such as a gay resort, for instance. Additionally, some reports presented secondary analyses of big data. This diversity suggests the numerous technological possibilities for addressing health concerns, particularly for nonclinical and public health purposes. Hence, for future research we also suggest to consider a more in-depth quantitative exploration of how telehealth mitigated or failed to address health care gaps, particularly in clinical areas like hormone therapy delays and mental health support or in time frames such as pandemics.

Belonging to a gender minority group is likely to activate different stressors that are also relevant for health conditions and healthcare needs of different populations, and must be considered also for the use of telehealth tools (Frost et al., 2015). Thus, there is a crucial need for more focused and comprehensive research that encompasses diverse transgender-populations and encompasses various practices, enabling a more informed approach to developing suitable offerings for this community.

### 4.1 Limitations

It is important to acknowledge the limitations of this study. The findings of the current review might not be generalized to all transgender-persons or all healthcare contexts, as our search strategy in terms of language and search terms focused on specific populations or settings and did not represent the full spectra of cultural understandings of transgender-persons, such as the Samoan fa’afafine (Poasa, 1992). This limitation in our search strategy might have led to an overrepresentation of geographical regions, such as US and Canada. Their particular healthcare contexts might differ from underrepresented healthcare contexts in terms of access to the internet, of access to healthcare in general as well as to gender-affirming healthcare. However, we included all age groups to provide a comprehensive overview that considers the various experiences and challenges across different age demographics within the transgender-community. Our database selection might have excluded further records in literature search. For instance, we did not consider the database PsycInfo, as we wanted to focus on the relation between transgender-persons and the technological aspect of telehealth research. Our search strategy was also limited to a specific timeframe (2000–2021) and may have missed more recent studies that were not available at the time of the search. Our study only analyzed peer-reviewed articles, which may have excluded relevant research in other types of publications, such as conference proceedings, books, or reports.

Notably, this review did not address ways how telehealth advances transgender-persons’ healthcare needs and inequities. Herein, we rather summarized the current evidence on transgender-individuals’ health issues discussed in research on telehealth use, which we estimated to be a relevant aspect for future research, including systematic literature reviews.

## 5 Conclusion

The telehealth tools we analyzed during our scoping review primarily discussed health issues that were also discussed to be impacted by the COVID-19 pandemic. Some tools also focused on the lack of knowledge among healthcare providers, who may need education and training on the diversity of non-binary groups and appropriate care for gender affirming treatments. Although telehealth solutions for transgender-persons may attempt to be inclusive of diverse groups of transgender-persons and make efforts to address the specific health issues of transgender-men and transgender-women, a risk of objectifying transgender-persons exists, in particular transgender-women. Future research should focus on intersectionality in transgender-persons’ experiences without reducing to sexual practices for instance. In this regard, investigating the interactions between technology and transgender-persons, as well as including groups outside of the healthcare system, might help to advance the development of telehealth tools for transgender-persons and explore possibilities of an adaptive use of existing tools.

## References

[ref1] Akinola M. Wirtz A. L. Chaudhry A. Cooney E. Reisner S. L. (2021). Perceived acceptability and feasibility of HIV self-testing and app-based data collection for HIV prevention research with transgender women in the United States. AIDS Care 33, 1079–1087. doi: 10.1080/09540121.2021.1874269, PMID: 33487032 PMC8298585

[ref2] Albury K. Dietzel C. Pym T. Vivienne S. Cook T. (2021). Not your unicorn: trans dating app users’ negotiations of personal safety and sexual health. Health Soc Rev. 30, 72–86. doi: 10.1080/14461242.2020.1851610, PMID: 33622202

[ref3] Asaad M. Rajesh A. Vyas K. Morrison S. D. (2020). Telemedicine in transgender care: a twenty-first-century beckoning. Plast. Reconstr. Surg. 146, 108e–109e. doi: 10.1097/PRS.0000000000006935, PMID: 32590687

[ref4] Benkeser D. Horvath K. Reback C. J. Rusow J. Hudgens M. (2020). Design and analysis considerations for a sequentially randomized HIV prevention trial. Stat. Biosci. 12, 446–467. doi: 10.1007/s12561-020-09274-3, PMID: 33767798 PMC7986973

[ref5] Blosnich J. R. Rodriguez K. L. Hruska K. L. Kavalieratos D. Gordon A. J. Matza A.. (2019). Utilization of the veterans affairs' transgender e-consultation program by health care providers: mixed-methods study. JMIR Med. Inform. 7:e11695. doi: 10.2196/11695 31344672 PMC6682290

[ref6] Braun V. Clarke V. (2006). Using thematic analysis in psychology. Qual. Res. Psychol. 3, 77–101. doi: 10.1191/1478088706qp063oa

[ref7] Braun V. Clarke V. (2014). What can “thematic analysis” offer health and wellbeing researchers? Int. J. Qual. Stud. Health Well Being 9:26152. doi: 10.3402/qhw.v9.26152, PMID: 25326092 PMC4201665

[ref8] Coleman E. Radix A. E. Bouman W. P. Brown G. R. de Vries A. L. C. Deutsch M. B.. (2022). Standards of Care for the Health of transgender and gender diverse people, version 8. Int. J. Transgend. Health 23, S1–S259. doi: 10.1080/26895269.2022.2100644, PMID: 36238954 PMC9553112

[ref9] Craig S. L. Iacono G. Pascoe R. Austin A. (2021). Adapting clinical skills to telehealth: applications of affirmative cognitive-behavioral therapy with LGBTQ+ youth. Clin. Soc. Work. J. 49, 471–483. doi: 10.1007/s10615-021-00796-x, PMID: 33678921 PMC7922718

[ref10] Craig S. L. Leung V. W. Y. Pascoe R. Pang N. Iacono G. Austin A.. (2021). Affirm online: Utilising an affirmative cognitive–behavioural digital intervention to improve mental health, access, and engagement among LGBTQA+ youth and young adults. Int. J. Environ. Res. Public Health 18, 1–18. doi: 10.3390/ijerph18041541, PMID: 33562876 PMC7915123

[ref9021] Crenshaw K. (1989). Demarginalizing the intersection of race and sex: A black feminist critique of antidiscrimination doctrine, feminist theory and antiracist politics. Univ. Chic. Leg. Forum 1, 139–167.

[ref11] da Silva C. E. Smith L. R. Patterson T. L. Semple S. J. Harvey-Vera A. Nunes S.. (2020). Stigma and web-based sex seeking among men who have sex with men and transgender women in Tijuana, Mexico: cross-sectional study. JMIR Publ. Heal Surveil. 6:e14803. doi: 10.2196/14803, PMID: 32031963 PMC7055757

[ref12] D'Angelo A. B. Argenio K. Westmoreland D. A. Appenroth M. N. Grov C. (2021). Health and access to gender-affirming care during COVID-19: experiences of transmasculine individuals and men assigned female sex at birth. Am. J. Mens Health 15:15579883211062681. doi: 10.1177/15579883211062681, PMID: 34861796 PMC8646200

[ref13] Day S. Smith J. Perera S. Jones S. Kinsella R. (2021). Beyond the binary: sexual health outcomes of transgender and non-binary service users of an online sexual health service. Int. J. STD AIDS 32, 896–902. doi: 10.1177/0956462420982830 34106795

[ref14] de Vere H. I. Linos E. (2022). Social Media for Public Health: framework for social media–based public health campaigns. J. Med. Internet Res. 24:e42179. doi: 10.2196/42179, PMID: 36515995 PMC9798262

[ref15] Doraiswamy S. Abraham A. Mamtani R. Cheema S. (2020). Use of telehealth during the COVID-19 pandemic: scoping review. J. Med. Internet Res. 22:e24087. doi: 10.2196/24087, PMID: 33147166 PMC7710390

[ref16] Dubov A. Fraenkel L. Goldstein Z. Arroyo H. McKellar D. Shoptaw S. (2021). Development of a smartphone app to predict and improve rates of suicidal ideation among transgender persons (TransLife): a qualitative study. J. Med. Internet Res. 23:e24023. doi: 10.2196/24023, PMID: 33596181 PMC8074983

[ref17] Fraser L. (2009). Etherapy: ethical and clinical considerations for version 7 of the world professional association for transgender health's standards of care. Int. J. Transgend. 11, 247–263. doi: 10.1080/15532730903439492

[ref18] Frost D. M. Lehavot K. Meyer I. H. (2015). Minority stress and physical health among sexual minority individuals. J. Behav. Med. 38, 1–8. doi: 10.1007/s10865-013-9523-8, PMID: 23864353 PMC3895416

[ref19] Gahbauer S. (2015). “Body images in medical teaching: A gender sensitive use” in Gender summit Europe 7 - mastering gender in research performance, contexts, and outcomes (Berlin: Gender Summit Europe).

[ref20] Gahbauer S. Voice work as a public health tool for trans* persons. Sustainable Health - 22nd Conference of the Austrian Society for Public Health (ÖGPH); (2019), Austria: Austrian Society for Public Health (ÖGPH).

[ref21] Gahbauer S. Haluza D. (2022). eHealth solutions for trans* persons: a systematic literature review of research from 2000 till 2021. Eur. J. Pub. Health 32:ckac131.508. doi: 10.1093/eurpub/ckac131.508

[ref22] Garcia M. M. Christopher N. A. De Luca F. Spilotros M. Ralph D. J. (2014). Overall satisfaction, sexual function, and the durability of neophallus dimensions following staged female to male genital gender confirming surgery: the Institute of Urology, London U.K. experience. Transl. Androl. Urol. 3, 156–162. doi: 10.3978/j.issn.2223-4683.2014.04.10, PMID: 26816764 PMC4708164

[ref23] Gava G. Fisher A. D. Alvisi S. Mancini I. Franceschelli A. Seracchioli R.. (2021). Mental health and endocrine telemedicine consultations in transgender subjects during the COVID-19 outbreak in Italy: a cross-sectional web-based survey. J. Sex. Med. 18, 900–907. doi: 10.1016/j.jsxm.2021.03.009, PMID: 33903046 PMC10016818

[ref24] Haluza D. Hofer F. (2020). Exploring perceptions on medical app use in clinical communication among Austrian physicians: results of a validation study. Health Informatics J. 26, 1659–1671. doi: 10.1177/1460458219888420 32723170

[ref25] Haluza D. Jungwirth D. (2018). ICT and the future of healthcare: aspects of pervasive health monitoring. Inform. Health Soc. Care 43, 1–11. doi: 10.1080/17538157.2016.1255215, PMID: 28005444

[ref26] Haluza D. Saustingl M. Halavina K. (2020). Perceptions of practitioners on telehealth and app use for smoking cessation and COPD care—an exploratory study. Medicina 56:698. doi: 10.3390/medicina56120698, PMID: 33333856 PMC7765310

[ref27] Hammarström A. Hensing G. (2018). How gender theories are used in contemporary public health research. Int. J. Equity Health 17:34. doi: 10.1186/s12939-017-0712-x, PMID: 29554916 PMC5859645

[ref28] Hawley J. L. Hancock A. B. (2021). Incorporating Mobile app Technology in Voice Modification Protocol for transgender women. J. Voice 7:21. doi: 10.21037/mhealth-20-43, PMID: 34706847

[ref29] Hertling S. Hertling D. Martin D. Graul I. (2021). Acceptance, use, and barriers of telemedicine in transgender health Care in Times of SARS-CoV-2: Nationwide cross-sectional survey. JMIR Public Health Surveill. 7:e30278. doi: 10.2196/30278, PMID: 34591783 PMC8647970

[ref30] Holloway I. W. Jordan S. P. Dunlap S. L. Ritterbusch A. Reback C. J. (2020). Leveraging social networks and technology for HIV prevention and treatment with transgender women. AIDS Educ. Prev. 32, 83–101. doi: 10.1521/aeap.2020.32.2.83, PMID: 32539480 PMC7709895

[ref31] Jarrett B. A. Peitzmeier S. M. Restar A. Adamson T. Howell S. Baral S.. (2021). Gender-affirming care, mental health, and economic stability in the time of COVID-19: a multi-national, cross-sectional study of transgender and nonbinary people. PLoS One 16:e0254215. doi: 10.1371/journal.pone.0254215, PMID: 34242317 PMC8270151

[ref32] Kauth M. R. Shipherd J. C. Lindsay J. A. Kirsh S. Knapp H. Matza L. (2015). Teleconsultation and training of VHA providers on transgender care: implementation of a multisite hub system. Telemed. J. E Health 21, 1012–1018. doi: 10.1089/tmj.2015.0010, PMID: 26171641

[ref33] Kirby T. (2016). Sari Reisner - making transgender health visible. Lancet 388:332. doi: 10.1016/S0140-6736(16)30839-X, PMID: 27323920

[ref34] Kuhns L. M. Hereth J. Garofalo R. Hidalgo M. Johnson A. K. Schnall R.. (2021). A uniquely targeted, Mobile app-based HIV prevention intervention for young transgender women: adaptation and usability study. J. Med. Internet Res. 23:e21839. doi: 10.2196/21839, PMID: 33787503 PMC8047777

[ref35] Lloyd C. E. M. Finn M. D. (2017). Authenticity, validation and sexualisation on Grindr: an analysis of trans women’s accounts. Psychol. Sex. 8, 158–169. doi: 10.1080/19419899.2017.1316769

[ref36] MacCarthy S. Reisner S. L. Nunn A. Perez-Brumer A. Operario D. (2015). The time is now: attention increases to transgender health in the United States but scientific knowledge gaps remain. LGBT Health 2, 287–291. doi: 10.1089/lgbt.2014.0073, PMID: 26788768 PMC4716649

[ref37] MacKinnon J. Bremshey A. (2020). Perspectives from a webinar: COVID-19 and sexual and reproductive health and rights. Sex Reprod. Health Matters 28:1763578. doi: 10.1080/26410397.2020.1763578, PMID: 32354272 PMC7888038

[ref38] Magnus M. Edwards E. Dright A. Gilliam L. Brown A. Levy M.. (2020). Development of a telehealth intervention to promote care-seeking among transgender women of color in Washington, DC. Public Health Nurs. 37, 262–271. doi: 10.1111/phn.12709, PMID: 32017202

[ref39] Masullo G. Coppola M. (2021). Scripts and sexual Markets of Transgender People on online dating apps: a Netnographic study. Ital Sociol Rev. 11, 319–341. doi: 10.13136/isr.v11i4S.437

[ref40] McGregor K. Williams C. R. Botta A. Mandel F. Gentile J. (2023). Providing essential gender-affirming telehealth services to transgender youth during COVID-19: a service review. J. Telemed. Telecare 29, 147–152. doi: 10.1177/1357633X221095785, PMID: 35570726 PMC9117984

[ref41] McQuoid J. Thrul J. Lopez-Paguyo K. Ling P. M. (2021). Exploring multiple drug use by integrating mobile health and qualitative mapping methods - an individual case study. Int. J. Drug Policy 97:103325. doi: 10.1016/j.drugpo.2021.103325, PMID: 34175527 PMC8585680

[ref42] Mintz L. J. Gillani B. Moore S. E. (2022). Telehealth in trans and gender diverse communities: the impact of COVID-19. Curr. Obstet. Gynecol. Rep. 11, 75–80. doi: 10.1007/s13669-022-00334-7 35463051 PMC9016376

[ref43] Mol A. Law J. (2004). Embodied action, enacted bodies: the example of Hypoglycaemia. Body Soc. 10, 43–62. doi: 10.1177/1357034x04042932

[ref44] Monaghesh E. Hajizadeh A. (2020). The role of telehealth during COVID-19 outbreak: a systematic review based on current evidence. BMC Public Health 20, 1193–1199. doi: 10.1186/s12889-020-09301-4, PMID: 32738884 PMC7395209

[ref45] Naszay M. Stockinger A. Jungwirth D. Haluza D. (2018). Digital age and the public eHealth perspective: prevailing health app use among Austrian internet users. Inform. Health Soc. Care 43, 390–400. doi: 10.1080/17538157.2017.1399131, PMID: 29256715

[ref46] Page M. J. McKenzie J. E. Bossuyt P. M. Boutron I. Hoffmann T. C. Mulrow C. D.. (2021). The PRISMA 2020 statement: an updated guideline for reporting systematic reviews. BMJ 372:n71. doi: 10.1136/bmj.n71, PMID: 33782057 PMC8005924

[ref47] Pankey T. L. Heredia D. Jr. Vencill J. A. Gonzalez C. A. (2021). Gender-affirming telepsychology during and after the COVID-19 pandemic: recommendations for adult transgender and gender diverse populations. J. Health Serv. Psychol. 47, 181–189. doi: 10.1007/s42843-021-00048-z, PMID: 34693297 PMC8520334

[ref48] Perry Y. Strauss P. Lin A. (2018). Online interventions for the mental health needs of trans and gender diverse young people. Lancet Psychiatry 5:e6. doi: 10.1016/S2215-0366(18)30017-8, PMID: 29413141

[ref49] Pinna F. Paribello P. Somaini G. Corona A. Ventriglio A. Corrias C.. (2022). Mental health in transgender individuals: a systematic review. Int. Rev. Psychiatry 34, 292–359. doi: 10.1080/09540261.2022.2093629 36151828

[ref50] Poasa K. (1992). The Samoan fa'afafine: one case study and discussion of transsexualism. J. Psychol. Hum. Sex. 5, 39–51. doi: 10.1300/J056v05n03_04

[ref51] Potapov A. Olayiwola J. N. Radix A. E. Meacher P. Sajanlal S. Gordon A. (2021). Electronic consultations as an educational tool to improve the care of transgender patients in primary care. J. Health Care Poor Underserved 32, 680–687. doi: 10.1353/hpu.2021.0097, PMID: 34120969

[ref52] Puckett J. A. Matsuno E. Dyar C. Mustanski B. Newcomb M. E. (2019). Mental health and resilience in transgender individuals: what type of support makes a difference? J. Fam. Psychol. 33, 954–964. doi: 10.1037/fam0000561, PMID: 31318262 PMC7390536

[ref53] Radix A. E. Bond K. Carneiro P. B. Restar A. (2022). Transgender individuals and digital health. Curr. HIV/AIDS Rep. 19, 592–599. doi: 10.1007/s11904-022-00629-7, PMID: 36136217 PMC9493149

[ref54] Reback C. J. Rusow J. A. Cain D. Benkeser D. Arayasirikul S. Hightow-Weidman L.. (2020). Technology-based stepped care to stem transgender adolescent risk transmission: protocol for a randomized controlled trial (TechStep). JMIR Res. Protoc. 9:e18326. doi: 10.2196/18326, PMID: 32788149 PMC7458064

[ref55] Reisner S. L. Choi S. K. Herman J. L. Bockting W. Krueger E. A. Meyer I. H. (2023). Sexual orientation in transgender adults in the United States. BMC Public Health 23:1799. doi: 10.1186/s12889-023-16654-z, PMID: 37715161 PMC10503109

[ref56] Reisner S. L. Poteat T. Keatley J. Cabral M. Mothopeng T. Dunham E.. (2016). Global health burden and needs of transgender populations: a review. Lancet 388, 412–436. doi: 10.1016/S0140-6736(16)00684-X, PMID: 27323919 PMC7035595

[ref57] Restar A. J. Jin H. Jarrett B. Adamson T. Baral S. D. Howell S.. (2021). Characterising the impact of COVID-19 environment on mental health, gender affirming services and socioeconomic loss in a global sample of transgender and non-binary people: a structural equation modelling. BMJ Glob. Health 6:e004424. doi: 10.1136/bmjgh-2020-004424, PMID: 33753401 PMC7985976

[ref58] Rogers C. Webberley M. Mateescu R. El Rakhawy Y. Daly-Gourdialsing A. Webberley H. (2020). A retrospective study of positive and negative determinants of gamete storage in transgender and gender-diverse patients. Int. J. Transgend. Health 22, 167–178. doi: 10.1080/26895269.2020.1848693, PMID: 34961859 PMC8040686

[ref59] Ross K. A. Castle B. G. (2017). A culture-centered approach to improving healthy trans-patient-practitioner communication: recommendations for practitioners communicating withTrans individuals. Health Commun. 32, 730–740. doi: 10.1080/10410236.2016.1172286, PMID: 27399644

[ref60] Roy J. Levy D. R. Senathirajah Y. (2022). Defining telehealth for research, implementation, and equity. J. Med. Internet Res. 24:e35037. doi: 10.2196/35037, PMID: 35416778 PMC9047847

[ref61] Rudin J. Billing T. Farro A. Yang Y. (2023). When are trans women treated worse than trans men? Equal. Divers. Inclus. 42, 723–736. doi: 10.1108/EDI-08-2021-0195

[ref62] Russell M. R. Rogers R. L. Rosenthal S. M. Lee J. Y. (2022). Increasing access to Care for Transgender/gender diverse youth using telehealth: a quality improvement project. Telemed. J. E Health 28, 847–857. doi: 10.1089/tmj.2021.0268, PMID: 34637658 PMC9231660

[ref63] Sell R. Goldberg S. Conron K. (2015). The utility of an online convenience panel for reaching rare and dispersed populations. PLoS One 10:e0144011. doi: 10.1371/journal.pone.0144011, PMID: 26641840 PMC4671660

[ref64] Sequeira G. M. Kidd K. M. Coulter R. W. S. Miller E. Fortenberry D. Garofalo R.. (2020). Transgender Youths' perspectives on telehealth for delivery of gender-affirming care. J. Adolesc. Health 68, 1207–1210. doi: 10.1016/j.jadohealth.2020.08.028, PMID: 32980246 PMC7510534

[ref65] Sharma A. Kahle E. Todd K. Peitzmeier S. Stephenson R. (2019). Variations in testing for HIV and other sexually transmitted infections across gender identity among transgender youth. Transgend. Health 4, 46–57. doi: 10.1089/trgh.2018.0047, PMID: 30805557 PMC6386078

[ref66] Shipherd J. C. Kauth M. R. Matza A. (2016). Nationwide interdisciplinary E-consultation on transgender Care in the Veterans Health Administration. Telemed. J. E Health 22, 1008–1012. doi: 10.1089/tmj.2016.0013, PMID: 27159795

[ref67] Silva C. Fung A. Irvine M. A. Ziabakhsh S. Hursh B. E. (2021). Usability of virtual visits for the routine clinical Care of Trans Youth during the COVID-19 pandemic: youth and caregiver perspectives. Int. J. Environ. Res. Public Health 18:11321. doi: 10.3390/ijerph182111321, PMID: 34769838 PMC8583569

[ref68] Singh J. Lou A. Green M. Keely E. Greenaway M. Liddy C. (2021). Evaluation of an electronic consultation service for transgender care. BMC Fam. Pract. 22:55. doi: 10.1186/s12875-021-01401-3, PMID: 33743596 PMC7980551

[ref69] Smith A. J. Hallum-Montes R. Nevin K. Zenker R. Sutherland B. Reagor S.. (2018). Determinants of transgender individuals’ well-being, mental health, and suicidality in a rural state. J. Rural Ment. Health 42, 116–132. doi: 10.1037/rmh0000089, PMID: 30333896 PMC6186454

[ref70] Stephenson R. Todd K. Kahle E. Sullivan S. P. Miller-Perusse M. Sharma A.. (2020). Project moxie: results of a feasibility study of a telehealth intervention to increase HIV testing among binary and nonbinary transgender youth. AIDS Behav. 24, 1517–1530. doi: 10.1007/s10461-019-02741-z, PMID: 31760536 PMC7162704

[ref71] Stryker S. Currah P. Moore L. J. (2008). Introduction: trans-, trans, or transgender? Women's Stud. Q. 36, 11–22. doi: 10.1353/WSQ.0.0112

[ref72] Sun C. J. Anderson K. M. Kuhn T. Mayer L. Klein C. H. (2020). A sexual health promotion app for transgender women (trans women connected): development and usability study. JMIR Mhealth Uhealth 8:e15888. doi: 10.2196/15888, PMID: 32396131 PMC7251477

[ref73] Sun C. J. Anderson K. M. Mayer L. Kuhn T. Klein C. H. (2019). Findings from formative research to develop a strength-based HIV prevention and sexual health promotion mHealth intervention for transgender women. Transgend. Health 4, 350–358. doi: 10.1089/trgh.2019.0032, PMID: 32042925 PMC6931010

[ref74] TGEU (Trans Europe and Central Asia). (2024). Intersectionality. Available at: https://www.tgeu.org/topics/trans-intersectionality/ (Accessed October 28, 2024).

[ref75] Tricco A. C. Lillie E. Zarin W. O'Brien K. K. Colquhoun H. Levac D.. (2018). PRISMA extension for scoping reviews (PRISMA-ScR): checklist and explanation. Ann. Intern. Med. 169, 467–473. doi: 10.7326/M18-0850, PMID: 30178033

[ref76] van der Miesen A. I. R. Raaijmakers D. van de Grift T. C. (2020). “You have to wait a little longer”: transgender (mental) health at risk as a consequence of deferring gender-affirming treatments during COVID-19. Arch. Sex. Behav. 49, 1395–1399. doi: 10.1007/s10508-020-01754-3, PMID: 32519279 PMC7282831

[ref77] Wang Y. Pan B. Liu Y. Wilson A. Ou J. Chen R. (2020). Health care and mental health challenges for transgender individuals during the COVID-19 pandemic. Lancet Diabetes Endocrinol. 8, 564–565. doi: 10.1016/S2213-8587(20)30182-0, PMID: 32445629 PMC7239622

[ref78] Wernhart A. Gahbauer S. Haluza D. (2019). eHealth and telemedicine: practices and beliefs among healthcare professionals and medical students at a medical university. PLoS One 14:e0213067. doi: 10.1371/journal.pone.0213067, PMID: 30818348 PMC6394957

[ref79] Wirtz A. L. Cooney E. E. Stevenson M. Radix A. Poteat T. Wawrzyniak A. J.. (2021). Digital epidemiologic research on multilevel risks for HIV acquisition and other health outcomes among transgender women in eastern and southern United States: protocol for an online cohort. JMIR Res. Protoc. 10:e29152. doi: 10.2196/29152, PMID: 33900202 PMC8111508

[ref80] World Health Organisation (2010). Telemedicine: Opportunities and developments in member states: Report on the second global survey on eHealth. Geneva: World Health Organisation.

[ref9022] World Health Organization (WHO). (2024). Gender incongruence and transgender health in the ICD. Available at: https://www.who.int/standards/classifications/frequently-asked-questions/gender-incongruence-and-transgender-health-in-the-icd (Accessed June 17, 2024).

